# Obstructive Sleep Apnea and Auditory Dysfunction—Does Snoring Sound Play a Role?

**DOI:** 10.3390/diagnostics12102374

**Published:** 2022-09-30

**Authors:** Chun-Ting Lu, Li-Ang Lee, Guo-She Lee, Hsueh-Yu Li

**Affiliations:** 1 Department of Otolaryngology-Head and Neck Surgery, New Taipei Municipal Tucheng Hospital (Built and Operated by Chang Gung Medical Foundation), New Taipei City 23652, Taiwan; 2 Department of Otorhinolaryngology-Head and Neck Surgery, Linkou Chang Gung Memorial Hospital, Taoyuan City 333, Taiwan; 3 Faculty of Medicine, Chang Gung University, Taoyuan City 33302, Taiwan; 4 Faculty of Medicine, School of Medicine, National Yang-Ming University, Taipei 112, Taiwan; 5 Department of Otolaryngology, Taipei City Hospital, Ren-Ai Branch, Taipei 10629, Taiwan

**Keywords:** obstructive sleep apnea, snoring, auditory dysfunction, tinnitus, intra-ear canal snoring sound energy

## Abstract

The objective of the study was to investigate the relationship between obstructive sleep apnea (OSA) and auditory dysfunction, and to clarify the role of snoring sounds in contributing to auditory dysfunction. A comprehensive assessment of OSA and the auditory system was performed, including overnight polysomnography, detection of the intra-ear canal snoring sound energy (SSE), pure tone average (PTA), tinnitus pitch matching, the tinnitus handicap inventory (THI), and the Epworth sleepiness scale (ESS). The patients were identified as having tinnitus if their THI score was higher than zero or their tinnitus pitches were matched to specific frequencies. The median age, body mass index, and apnea–hypopnea index score were 41 years, 26.4 kg/m^2^, and 29.9 events/h, respectively. Among the 50 participants, 46 (92%) had a normal PTA, and only 4 (8%) patients had mild hearing loss. There was no significant difference in PTA among OSA severities (*p* = 0.52). Among the 50 participants, 33 patients (66%) were identified as having tinnitus. In the tinnitus group (n = 33), the ESS score (*p* = 0.01) and intra-ear canal SSE of 851–1500 Hz (*p* = 0.04) were significantly higher than those in the non-tinnitus group (n = 17). OSA patients with a higher ESS score had a higher risk of tinnitus (odds ratio 1.22 [95% CI: 1.01–1.46]). OSA-related auditory dysfunction emerged in tinnitus rather than in hearing impairment. OSA patients with daytime sleepiness had a higher risk of tinnitus. High-frequency SSE can jeopardize cochlea and is a potential mechanism contributing to tinnitus. Detection of snoring sounds through an intra-ear canal device may be more precise in assessing acoustic trauma from snoring sounds to vulnerable auditory system and thus warrants further research.

## 1. Introduction

Obstructive sleep apnea (OSA) is closely related to auditory dysfunction, including hearing impairment [1,2,3], tinnitus [1,4], vestibular dysfunction [5], and even sudden hearing loss [6]. OSA may cause auditory dysfunction via multiple mechanisms, including blood hyperviscosity [7], intermittent hypoxemia [8], oxidative stress1, and changes in sympathetic tone [9]. However, there is no solid consensus regarding how OSA influences the auditory system. According to our previous study [10], snoring sounds may cause carotid wall thickening in OSA patients, and there have been few studies investigating the influence of snoring sounds on the auditory system. In the study, we proposed a novel device to detect snoring sounds through the ear canal because it is closer to the auditory system than other related locations and more likely to reflect acoustic trauma to the vulnerable auditory system from snoring sound energy (SSE). The purposes of this study were to (1) investigate the relationship of polysomnographic parameters and auditory dysfunction, and (2) clarify the role of snoring sounds in auditory dysfunction.

## 2. Materials and Methods

### 2.1. Study Design

We conducted a study project for a period of 2 years and tried to collect sixty subjects based on clinical feasibility that involved the capacity and waiting time of polysomnography, and the patient’s willingness to join the project and receive auditory function tests. The eligible subjects were assessed for auditory examinations, snoring sound analysis, and further statistical analysis. This study was approved by the Institutional Review Board of the Chang Gung Memorial Foundation (201601978A3) and supported by Ministry of Science and Technology Taiwan (NMRPG3G6302)

### 2.2. Setting

This study was conducted at the Department of Otorhinolaryngology Head and Neck Surgery at the Linkou-Chang Gung Memorial Hospital between 1 August 2017 and 31 July 2020.

### 2.3. Participants

We consecutively and prospectively recruited sixty adults. The inclusion criteria included: (1) age: 30–60 years old; (2) subjective symptoms of snoring and apnea–hypopnea index ≥ 5 events/h; (3) agreement to attend the study. 

**Figure 1:**
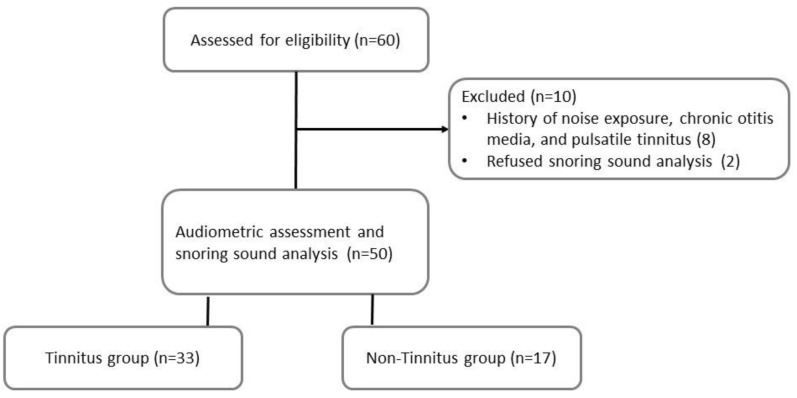
Flowchart of the study.

The exclusion criteria included: (1) body mass index (BMI) > 35 kg/m^2^; (2) history of sudden hearing loss, Meniere’s disease, noise exposure, ototoxic medication, chronic otitis media, mastoiditis, head trauma, or gastroesophageal reflux disease under medication; (3) chronic insomnia and psychiatric disorder; (4) objective tinnitus and pulsatile tinnitus. Eight patients were excluded due to a history of noise exposure, chronic otitis media, and pulsatile tinnitus. Two patients refused to participate in the snoring sound analysis (Figure 1). 

### 2.4. Polysomnography

A standard overnight polysomnography (Nicolet UltraSom System, Madison, WI, USA) was performed, including electrocardiography, electroencephalography, electro-oculography, submental and leg electromyography, and pulse oximetry. Airflow from the mouth and nose was detected by a thermistor and cannula. Respiratory movement was measured by inductive plethysmographic bands in the chest and abdominal wall. Snoring was recorded by a submental microphone. Video recording was used to assess the behavior of all participants while sleeping.

Apnea was defined as the cessation of airflow for at least 10 seconds. Hypopnea was defined as an abnormal respiratory event with at least a 30% reduction in airflow, as compared to baseline, lasting at least 10 seconds, and with at least 3% oxygen desaturation and/or an arousal, based on the American Academy of Sleep Medicine (AASM) 2012 manual. The apnea–hypopnea index (AHI) is the number of events of apnea plus hypopnea per hour of total sleep time. OSA was defined as obstructive AHI ≥ 5 events/h. Patients were further categorized as having mild (AHI ≥ 5), moderate (AHI ≥ 15 to <30), or severe (AHI ≥ 30) OSA [11].

### 2.5. Audiometric Assessment

The average thresholds of 500, 1k, 2k, and 4k Hz were calculated as the pure tone average (PTA) to represent the patient’s hearing. The PTA of the worse ear of each patient was used for further classification as: normal hearing (PTA ≦25 dB HL); mild hearing loss (PTA between 26 and 40 dB HL); moderate hearing loss (PTA between 41 and 60 dB HL); severe hearing loss (PTA between 61 and 80 dB HL); profound hearing loss (PTA ≧ 81 dB HL). All participants were asked if they had tinnitus or not, and those with subjective tinnitus were tested with the tinnitus handicap inventory (THI) questionnaire and tinnitus pitch matching. The patients were considered to have tinnitus (tinnitus group) if (1) the patient’s THI score was higher than zero, or (2) their tinnitus pitches were matched to a specific frequency; others were considered to belong to the non-tinnitus group.

### 2.6. Detection and Analysis of Snoring Sounds

**Figure 2:**
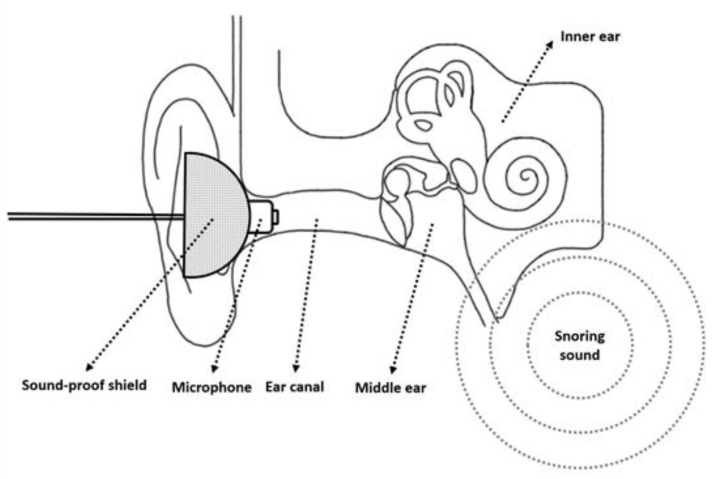
A microphone with a sound-proof shield was inserted into the ear canal for the recording of snoring sounds.

In our previous studies, the sound recorder was positioned 100 cm above the participant’s head to record snoring sounds during sleep [10]. In order to better understand the influence of snoring sound on the auditory system, we designed an intracanal microphone, which was placed near to the snoring sound source, to detect the snoring sound, (Figure 2).

A microphone with a soundproof shield was inserted into the ear canal, and the intra-ear canal snoring sound was recorded by a portable digital sound recorder (PCM-D50, Sony). The speaker produced 1000 Hz pure tones at an intensity of 94 dB in sound pressure level (SPL) in the sound-treated room, and these were used for intensity calibration of the sound recorder. The 1 min signals before the first snore were considered background noise, and the root mean square (RMS) was obtained as a baseline to distinguish snores from noise.

Snoring sound analysis was performed throughout each recording using a 0.25 s time window with no overlapping data. The signals of an analytic window with an energy at least 6 dB stronger than background noises were considered to be snore epochs; all others were considered noise epochs. Consecutive snore epochs lasting 0.5 to 3 s were considered snores [12]. The power spectrum was acquired for each window using fast Fourier transformation (0 to 1500 Hz) with a frequency resolution of 4.0 Hz. The energy levels (between snores and noise) of various frequency domains (in dB) were acquired using a specially developed software program called snoring sound energy [13]. For further analysis, the average snoring sound energy of three different frequency domains (B1, 4–300 Hz; B2, 301–850 Hz; B3, 851–1500 Hz) was calculated according to our previous study [14].

### 2.7. Statistical Analysis

According to the D’Agostino and Pearson normality test, most of the variables were found to have non-normal distribution, and the descriptive statistics of these variables were presented as the median and interquartile range (IQR). Comparisons of the PTA between mild, moderate, and severe OSA were made with the Kruskal–Wallis test. Comparisons of variables between the tinnitus and non-tinnitus groups were made with the Mann–Whitney U test. The differences in the proportions of tinnitus and OSA severity were tested using Fisher’s exact test. The Spearman correlation test was used to assess correlations between different parameters. Variables with a *p* value < 0.20 on univariate analysis were included in the multivariate logistic regression to find predictors of tinnitus. The ROC curve and the area under the ROC curve (AUROC) were used for validation of the regression model. All *p* values were two-sided, and statistical significance was accepted at *p* < 0.05. All statistical analyses were performed using IBM SPSS software (version 23; International Business Machines Corp., Armonk, NY, USA) and Graph Pad Prism 7.00 for Windows (Graph Pad Software Inc., San Diego, CA, USA).

## 3. Results

### 3.1. Study Population

Fifty patients completed the auditory assessment and snoring sound analysis. The median age of the participants was 41 years (IQR: 38–48 years). The median body mass index was 26.4 kg/m^2^ (IQR: 24.0–29.5 kg/m^2^). Among the fifty participants, forty-four (88%) patients were male; twelve (24%) patients had mild OSA, thirteen (26%) patients had moderate OSA, and twenty-five (50%) patients had severe OSA. The median AHI was 29.9 events/h (IQR: 17.2–48.1 events/h).

### 3.2. Hearing Thresholds

**Figure 3:**
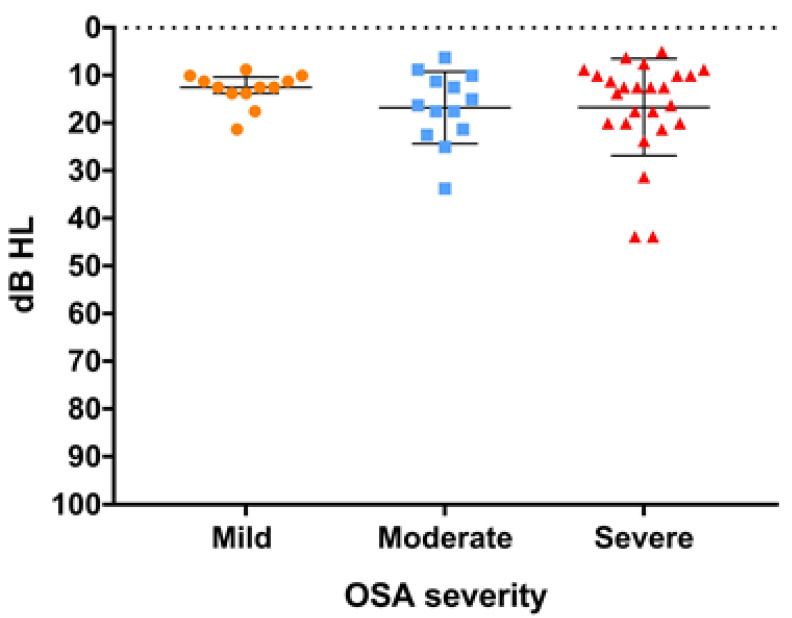
Pure tone average (PTA) of the worse ear is shown with scatter dot plot (line: median and interquartile range) and grouped (mild, moderate, severe) according to obstructive sleep apnea (OSA) severity. The difference in PTA between OSA severities showed no statistical significance. (*p* = 0.52).

Among the 50 patients, 46 (92%) had normal PTA and only 4 (8%) patients had mild hearing loss (Figure 3). As the PTA was divided into groups based on OSA severity, the median PTA was 12.5 dB (IQR: 10.3–13.8 dB) in mild OSA, 16.3 dB (IQR: 10.6–21.9 dB) in moderate OSA, and 12.5 dB (10.2–20.0 dB) in severe OSA. There was no significant difference in PTA between OSA severities (*p* = 0.52) (Figure 3).

### 3.3. Tinnitus and Snoring Sound Energy

Among the 50 patients, 33 (66%) cases had tinnitus, and the median THI score was 6 (IOR: 2–19). A total of 25 cases completed pitch matching, and the tinnitus pitch of 13 cases was matched at 8000 Hz, while the others were matched at a frequency between 125 and 6000 Hz (Table 1). The ESS score was significantly higher in the tinnitus group than the non-tinnitus group (Mann–Whitney U test, *p* = 0.01), whereas no difference was found in age, BMI, AHI, or O2 saturation between the two groups (Table 1).

The proportion of patients with tinnitus and different OSA severities was tested with Fisher’s exact test, which showed no significant differences (*p* = 0.23) (Table 2).

**Figure 4:**
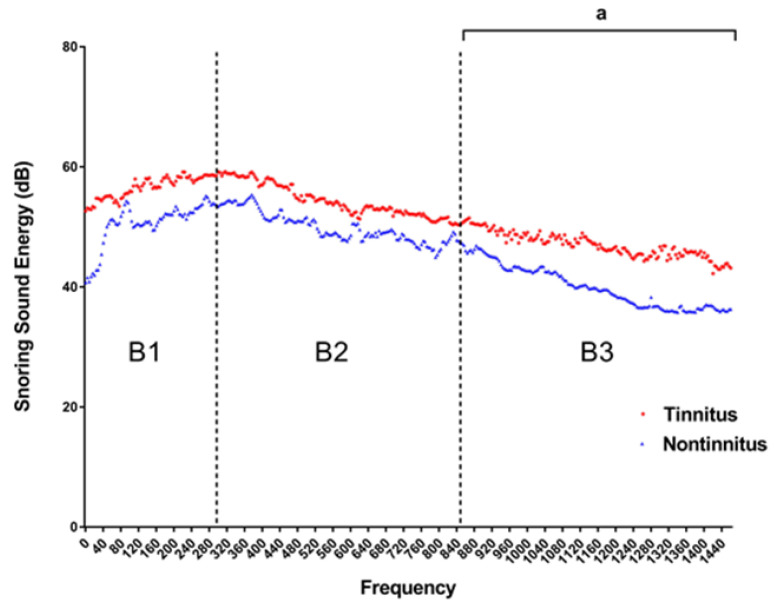
Comparisons of the intra-ear canal snoring sound energy (SSE) between tinnitus and non-tinnitus groups (n = 50). The SEE of B3 frequency domain was significantly higher in the tinnitus group than in the non-tinnitus group (*p* = 0.04). (Frequency domain, B1: 4–300 Hz; B2: 301–850 Hz; B3: 851–1500 Hz.). ^a^ Two-tailed *p* value < 0.05 (Mann–Whitney U test).

Figure 4 depicts the SSE of each frequency between the tinnitus group and non-tinnitus group. The snoring sound energy was significantly higher in the tinnitus group than in the non-tinnitus group at the B3 (851–1500 Hz) domain (Mann–Whitney U test, *p* value 0.04), while the snoring sound energy at B1 and B2 showed no significant differences (Table 3).

Univariate logistic analysis revealed that the Epworth sleepiness scale was a significant predictor of tinnitus, while the SEE of the B3 (851–1500 Hz) domain showed a marginal significance. In multivariate logistic regression, we found that the Epworth sleepiness scale was the only significant predictor of tinnitus in patients with OSA (Table 4). The risk of tinnitus rose as the Epworth sleepiness scale increased (OR = 1.22, CI = 1.01–1.46).

**Figure 5:**
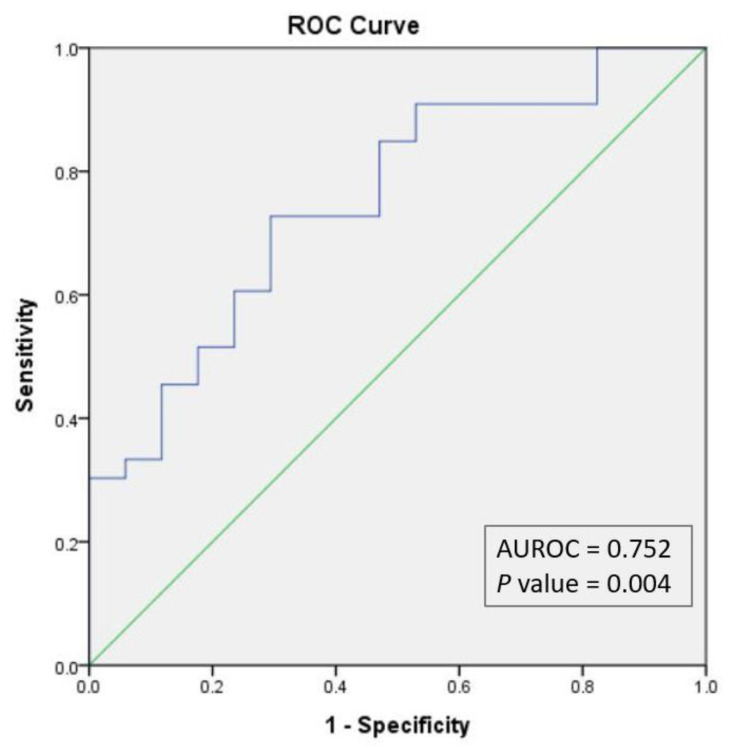
ROC curve for multivariate binary logistic regression. AUROC, area under the ROC curve.

As the validation procedure, we constructed the ROC curve for the probabilities estimated through the regression model. The AUROC = 0.752 (*p* value = 0.004) confirmed the efficiency of the model obtained (Figure 5).

## 4. Discussion

There are few studies discussing the role of OSA in the auditory system. Casale et al. found prolonged auditory brainstem response and lower DPOAE amplitude in patients with OSA [15]. Matsumura et al. also revealed lower amplitude of DPOAE in severe OSA, suggesting impairment of cochlear hair cells [16]. However, the results of OSA and hearing threshold are conflicting. Deniz et al. reported that patients with moderate and severe OSA had mild hearing loss compared to the mild OSA and control groups [2]. Furthermore, a population-based study by Chopra et al. showed that patients with sleep apnea had 30% higher odds of hearing impairment and a dose–response relationship between hearing impairment and OSA severity [9]. In contrast, Hwang et al. revealed that OSA did not affect the average hearing threshold, but had a negative association with PPS score in older subjects [3]. Martines et al. also revealed that there was no difference in the PTA between OSA and control groups, except for those patients with severe OSA, who had a higher threshold at extended frequencies (6k–16k Hz) [1]. Casale et al. revealed that although the mean hearing thresholds in OSA patients were significantly higher than in the control group, the hearing thresholds were still within normal limits [15]. In our study, only 8% patients suffered from mild hearing loss, and the PTA showed no significant difference between groups by OSA severity, which is compatible with the results of previous studies.

The estimated prevalence of tinnitus in adults is around 10% to 15% [17]. A systemic review showed that the prevalence of tinnitus ranged from 5.1% to 42.7% according to different age and criteria [18,19]. However, the prevalence of tinnitus in patients with OSA is not well-established. A recent population-based case–control study in Taiwan showed that the risk of tinnitus increased 1.36 times in middle-aged patients with sleep apnea [4]. Martines et al. also revealed that the prevalence of tinnitus in OSA patients was higher than in patients without OSA [1]. Although there was no control group (patients without OSA) in our study, the prevalence of tinnitus in OSA patients was 66%, which is higher than that in the general middle-aged population.

In our study, only 8% cases had mild hearing loss; in contrast, 66% cases had tinnitus. It seemed that the influence of OSA on the auditory system resulted more often in tinnitus than hearing impairment. In previous studies, OSA has been shown to influence the auditory system by multiple mechanisms, including blood hyperviscosity [7], intermittent hypoxemia [8], oxidative stress [1], and changes in sympathetic tone [9]. However, discussion about the relationship between OSA and tinnitus is rare in the current literature.

The mechanism of tinnitus is multifactorial, and tinnitus can arise from pathological changes along the auditory pathway [20]. Sleep disturbance was also proposed as one of the mechanisms of tinnitus formation [21]. Excessive daytime sleepiness is one of the symptoms in OSA, which is due to sleep fragmentation and hypoxemia [22], and the Epworth sleepiness scale is a common subjective method to access it. In our study, the Epworth sleepiness scale score was significantly higher in the tinnitus group, and patients had a higher risk of tinnitus as the score increased. In contrast, no significant difference was found in the AHI, the proportion of AHI severity, or O2 saturation between the tinnitus and non-tinnitus groups. Therefore, sleep disturbance due to sleep fragmentation may be one of the reasons that causes tinnitus in OSA. We should keep in mind that OSA patients may suffer from excessive daytime sleepiness and tinnitus at the same time.

In an animal study, tissue vibrations were observed to be one of the mechanisms that caused direct endothelial dysfunction and subsequent carotid atherosclerosis [23]. In a guinea pig model, the temporal bone vibration from an external electromagnetic shaker could cause threshold shift, and the hearing threshold was more vulnerable to higher-frequency vibrations [24]. Lee et al. and Chuang at al. also revealed that snoring sound energy may be related to common carotid artery intima–media injury in patients with OSA [10,14]. Furthermore, Ekin et al. found extended high-frequency hearing loss in OSA patients, and even in simple snorers [25]. Therefore, we hypothesized that tissue vibration damage may be another mechanism that influences the auditory system in patients with OSA.

In OSA patients, the palate and tongue base produce the vibration and snoring sound due to intermittent airway collapse during sleep. The vibration and snoring sounds are transmitted to the surrounding tissue, such as the carotid artery [10,26], and the auditory system. In this study, the intra-ear canal snoring sound energy of a tinnitus group was significantly higher than in the non-tinnitus group at the B3 (851–1500 Hz) domain. Therefore, we hypothesized that the vibration of high-frequency snoring sounds may cause tissue damage to the auditory organ and bring about subsequent tinnitus. This result is compatible with the previously mentioned guinea pig model [24], which showed that the auditory system was more vulnerable to higher-frequency vibrations.

There were several limitations of the present study. First, this small observational study lacked external validation. Second, there was no direct measurement of the vibration intensity. Third, there was no objective auditory assessment, such as auditory brainstem response, otoacoustic emission, or an extended high-frequency threshold test, conducted to support our hypothesis of the vibration damage in the auditory pathway. Fourth, the lack of exclusion of somatosensory tinnitus and other less common tinnitus conditions may have led to interpretation bias. Further study is warranted.

## 5. Conclusions

In this study, we tried to clarify the relationship between OSA and auditory dysfunction. We found that OSA brought about more tinnitus than hearing loss, and those patients with excessive daytime sleepiness had a higher risk of tinnitus. Furthermore, high-frequency snoring sounds, which are transmitted to the ear canal, may have a role in contributing to tinnitus.

## Figures and Tables

**Table 1:** Comparisons in demographic data, tinnitus data and polysomnographic data between tinnitus and non-tinnitus groups.

Variables	Tinnitus (n = 33)	Non-Tinnitus (n = 17)	*p* Value
*Demographic data*			
Age (years)	41 (37–48)	41 (38–46)	0.84
BMI (kg/m^2^)	26.4 (23.8–29.7)	26.3 (24.0–29.5)	0.97
*Tinnitus data*			
THI (0–100)	6 (2–19)	-	-
Pitch mapping (n, %)	25 (25/33, 75.8%)	-	-
8000 Hz (n, %)	13 (13/25, 52.0%)	-	-
125–6000 Hz (n, %)	12 (12/25, 48.0%)	-	-
*Polysomnography data*			
AHI (events/h)	31.9 (12.3–49.0)	24.9 (18.9–50.5)	0.81
Mean SpO2 (%)	93.0 (91.0–94.0)	94.0 (92.0–95.0)	0.37
Lowest SpO2 (%)	76.0 (65.0–79.0)	73.0 (71.5–82.0)	0.98
ESS (0–24)	11.0 (8.5–15)	8.0 (6.0–11.0)	0.01 ^a^

BMI, body mass index; THI, tinnitus handicap inventory; AHI, apnea–hypopnea index; ESS, Epworth sleepiness scale. All values are reported as median and interquartile ranges (IQRs). ^a^ Two-tailed *p* value < 0.05 (Mann–Whitney U test).

**Table 2:** Comparisons of OSA severity between tinnitus and non-tinnitus group.

	Tinnitus (n = 33)	Non-Tinnitus (n = 17)	*p* Value
OSA severity			0.23
Mild, n (%)	9 (27.3%)	3 (17.6%)	
Moderate, n (%)	6 (18.2%)	7 (41.2%)	
Severe, n (%)	18 (54.5%)	7 (41.2%)	

OSA, obstructive sleep apnea. The differences in the proportions of patients with tinnitus and OSA severity were tested using the Fisher’s exact test.

**Table 3:** Comparisons of snoring sound energy between tinnitus and non-tinnitus group.

	Tinnitus (n = 33)	Non-Tinnitus (n = 17)	*p* Value
SEE of B1 domain (dB)	55.7 (50.6–62.6)	53.0 (48.5–63.8)	0.43
SEE of B2 domain (dB)	54.4 (49.9–59.2)	51.5 (46.3–59.6)	0.36
SEE of B3 domain (dB)	46.8 (43.2–54.5)	42.1 (36.2–47.8)	0.04 ^a^

SEE, snoring sound energy; frequency domain, B1: 4–300 Hz; B2: 301–850 Hz; B3: 851–1500 Hz. All values are reported as median and interquartile ranges (IQRs). ^a^ Two-tailed *p* value < 0.05 (Mann–Whitney U test).

**Table 4:** Univariate and multivariate logistic regression models used to predict tinnitus.

Variables	Univariate Analysis		Multivariate Analysis	
	OR (95% CI)	*p* Value	OR (95% CI)	*p* Value
AHI	1.00 (0.97–1.03)	0.86	-	-
Mean SpO2	0.85 (0.63–1.13)	0.25	-	-
Lowest SpO2	0.05 (0.00–56.48)	0.40	-	-
Epworth sleepiness scale	1.25 (1.04–1.50)	0.02 ^a^	1.22 (1.01–1.46)	0.04 ^a^
SEE of B3 domain	1.09 (1.00–1.18)	0.045 ^a^	1.07 (0.99–1.16)	0.11

OR, odds ratio; CI, confidence intervals; AHI, apnea–hypopnea index; SEE, snoring sound energy; frequency domain, B1: 4–300 Hz; B2: 301–850 Hz; B3: 851–1500 Hz. ^a^ Two-tailed *p* value < 0.05 (logistic regression).

## Data Availability

The data are not publicly available due to the regulation of our institution and protection of patients’ privacy particular in small sample size group. However, the data presented in this study are available on request from the corresponding author for further research, if available.
